# A20 Attenuates the Fibrotic Response in the Trabecular Meshwork

**DOI:** 10.3390/ijms23041928

**Published:** 2022-02-09

**Authors:** Philip Mzyk, Emma G. Zalog, Colleen M. McDowell

**Affiliations:** Department of Ophthalmology and Visual Sciences, University of Wisconsin Madison, Madison, WI 53706, USA; pmzyk@wisc.edu (P.M.); zalog@wisc.edu (E.G.Z.)

**Keywords:** A20, trabecular meshwork, fibronectin, glaucoma

## Abstract

Although the extracellular matrix (ECM) in trabecular meshwork (TM) cells is known to be important in intraocular pressure (IOP) regulation, the molecular mechanisms involved in generating a glaucomatous environment in the TM are not completely understood. Recently we identified a molecular pathway, transforming growth factor beta 2 (TGFβ2)–toll-like receptor 4 (TLR4) signaling crosstalk, as an important regulator of glaucomatous damage in the TM, which contributes to fibrosis. Here we evaluate a novel molecular target, A20, also known as tumor necrosis factor alpha-induced protein 3 (TNFAIP3), which may help to block pathological TGFβ2–TLR4 signaling. Primary human TM cells were analyzed for A20 message and for A20 and fibronectin protein expression after treatment with TGFβ2. A20 message increased when the TLR4 pathway was inhibited in TM cells. In addition, TGFβ2, a known inducer of fibrosis, increased fibronectin expression, while at the same time decreasing the expression of A20. We then overexpressed A20 in TM cells in order to test the effect on treatment with TGFβ2, lipopolysaccharide (LPS), or cellular fibronectin extra domain A (cFN-EDA). Importantly, overexpression of A20 rescued the fibrotic response when TM cells were treated with TGFβ2, LPS, or cFN-EDA. In situ hybridization was used to probe for A20 RNA expression in age-matched control (C57BL/6J) mice and mice that constitutively express the EDA isoform of fibronectin (B6.EDA^+/+^). In this novel mouse model of glaucoma, A20 RNA was increased versus age-matched control mice in a cyclic manner at 6 weeks and 1 year of age, but not at 8 months. Overall, these data suggest that A20 may work through a negative feedback mechanism attenuating the ability of TGFβ2–TLR4 signaling to induce fibrosis.

## 1. Introduction

The glaucomas constitute a group of optic neuropathies with multiple clinical characteristics, including cupping of the optic disc and thinning and loss of the retinal nerve fiber layer [1]. Elevated intraocular pressure (IOP) is a large factor in the induction of glaucoma, and this stems from persistent damage to the trabecular meshwork (TM). This damage causes impairment in the ability of the aqueous humor to properly drain from the anterior segment. Specifically, changes in the extracellular matrix (ECM) surrounding the TM alter the drainage ability of the aqueous humor [2]. Clinically, glaucoma has been well-characterized, and several causative glaucoma genes have been identified. However, the molecular mechanisms responsible for the majority of cases remain to be fully elucidated. Transforming growth factor beta 2 (TGFβ2) signaling has been suggested to be involved in glaucomatous damage to the TM by affecting the ECM environment [3].

By utilizing our TGFβ2-induced ocular hypertension model, we have identified toll-like receptor 4 (TLR4) signaling as a novel molecular pathway involved in the development and progression of glaucomatous TM damage [3]. We demonstrated TGFβ2–TLR4 signaling crosstalk in the regulation of ECM and fibrogenesis in the TM as well as in the development of ocular hypertension [3]. Endogenous ligands of TLR4, known as damaged associated molecular patterns (DAMPs), such as fibronectin extra domain A (FN-EDA) have been shown to activate TLR4 and augment TGFβ signaling and downstream fibrotic responses [3,4]. Importantly, we have shown that constitutively active FN-EDA causes elevated IOP in mice [5]. Since the fibrotic response leads to the accumulation of more DAMPs, a feed-forward loop develops, leading to a further progression of the fibrotic response. Additionally, we have identified crosstalk between TGFβ2 and the transcription factor nuclear factor kappa beta (NF-κB) signaling pathways in the TM that contributes to glaucomatous ocular hypertension [6]. NF-κB is necessary for TGFβ2-induced ECM production and ocular hypertension [6]. The molecular mechanisms by which NF-κB upregulates the ECM are not fully understood, but our data suggest it is important for development of ocular hypertension and ECM changes in the TM.

Tumor necrosis factor-alpha-induced protein 3 (TNFAIP3), also known as A20, is a potent anti-inflammatory signaling molecule that restricts multiple intracellular signaling cascades [7]. Additionally, A20 is a ubiquitin-modifying enzyme that attenuates NF-κB signaling and TNF-mediated programmed cell death [7,8]. A20 knockout mice die prematurely by cachexia and multiorgan inflammation, which highlights the vital immunoregulatory and anti-inflammatory functions of A20 [9]. Most cells express only low amounts of A20 under basal conditions, but A20 expression is rapidly induced upon NF-κB activation due to the presence of two NF-κB binding sites in the A20 promoter [8]. Antigen receptor stimulation leads to a rapid decrease and subsequent reappearance of A20 in cells, suggesting that A20 removal upregulates NF-κB activation [8]. Overexpression of A20 in pancreatic islets of Langerhans confers resistance to cytokine-mediated activation of NF-κB, protecting them from apoptosis in the early post-transplantation period [10]. In lung, adenoviral delivery of A20 was shown to attenuate allergic airway inflammation through suppression of inflammatory cytokine production [11]. Genetic association studies have also identified linkage of SNPs in the A20 locus with susceptibility to fibrotic diseases such as scleroderma and methylation. Patterns in the A20 promoter region were found to be correlated with expression variability among glaucomatous donors [12]. 

Unchecked NF-κB signaling and persistent fibrosis in TM cells might result from impaired A20. A20’s two catalytic activities provide A20 with the capacity to regulate NF-κB signaling and have been shown to inhibit cellular apoptosis [8]. The inactivation of NF-κB may prevent further downstream signaling and block the pathogenetic fibrotic signaling associated with glaucoma. In addition, A20 has been linked to TGFβ2 signaling as A20 blocks intracellular TGFβ-SMAD signaling and attenuates fibrotic responses in other cell types [13]. However, A20 expression is known to be suppressed by TGFβ2 signaling, which could lead to a compounded effect on the TGFβ2–TLR4 feedforward loop [13]. Interestingly, vitamin E has been shown to increase A20 expression and subsequently attenuate NF-κB activity, and vitamin E treatment on TM cells in culture blocked TGFβ2-induced fibrotic responses [14,15]. These data suggest that pharmacologic or genetic rescue of A20 might restore appropriate regulation of fibrotic processes in the TM, leading to novel treatment options to lower IOP and further explaining the mechanisms involved in the development of glaucomatous TM damage.

In the present study, we demonstrate that inhibiting the TLR4 pathway with the selective TLR4 inhibitor TAK-242 increases the expression of A20 message, while TGFβ2 alone does not. Primary TM cells treated with TGFβ2 suppressed A20 expression, while at the same time exhibiting the classical increase in the ECM component fibronectin. Utilizing an Ad5 viral vector, we overexpressed A20 in primary TM cells and found that A20 overexpression rescues the fibrotic phenotype when TM cells are treated with TGFβ2, lipopolysaccharide (LPS) (a classical activator of the TLR4 pathway), and cFN-EDA (a DAMP activator of TLR4). Lastly, mice that constitutively express only FN containing EDA were found to have, when compared to age-matched control mice, increased A20 RNA levels at 6 weeks and 1 year, but not at 8 months of age. 

## 2. Results

### 2.1. Generation of Primary Human Trabecular Meshwork Cells

**Figure 1:**
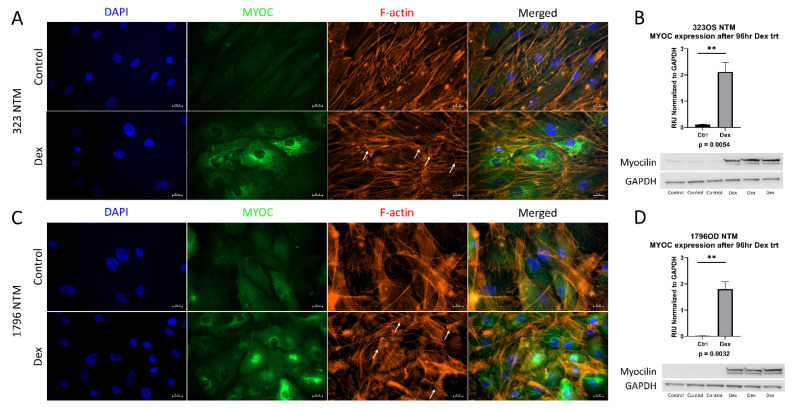
Characterization of primary trabecular meshwork (TM) cells. Primary TM cells were treated with dexamethasone (100 nM) for either 1 week (ICC analysis) or 96 h (western analysis). ICC analysis of both primary cell lines showed an induction of myocilin expression and CLAN formation, denoted by white arrows (**A**,**C**). Western blotting also showed an induction of myocilin expression after 4 days of dexamethasone treatment in both primary cell lines (**B**,**D**). GAPDH was used as a loading control. Statistical significance was determined by Student’s *t*-test, with *p* < 0.05 considered statistically significant. Error bars show SEM. ** = *p* ≤ 0.01.

All experiments were conducted with newly isolated and characterized primary human TM cells. According to established guidelines, primary human TM cell strains should induce myocilin expression upon dexamethasone treatment, as myocilin induction in response to dexamethasone is a reliable marker for TM cells, since neighboring ocular tissues and cells do not respond as robustly to this treatment [16]. Additionally, cross linked actin networks (CLAN) formation after dexamethasone treatment are another marker of TM cells [17]. Therefore, cells were treated with dexamethasone, and the induction of myocilin expression and CLAN formation was examined. In Figure 1A,C, both characterized lines show an induction of myocilin expression upon dexamethasone treatment, as well as the formation of CLANs. Myocilin increased after dexamethasone treatment, as assessed by immunoblot (Figure 1B,D). These data demonstrate that these new cell strains are primary human trabecular meshwork cells.

### 2.2. Selective Inhibition of the TLR4 Pathway Increases A20 mRNA Expression

**Figure 2:**
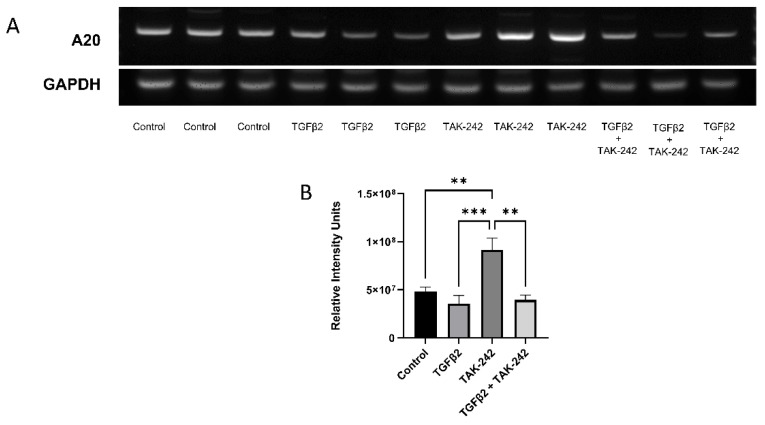
A20 gene expression in primary human TM cells increased with selective inhibition of the TLR4 pathway. (**A**) RT-PCR was performed after the indicated treatments with primers specific for human A20 and the housekeeping gene GAPDH. (**B**) A20 gene expression was significantly elevated by TAK-242 treatment. Statistical significance was determined by one-way ANOVA with Tukey post-hoc analysis, with *p* < 0.05 considered statistically significant. Error bars show SEM. ** = *p* ≤ 0.01, *** = *p* ≤ 0.001.

We have previously shown that selective inhibition of the TLR4 pathway with TAK-242 rescues the fibrotic response induced by TGFβ2 treatment in TM cells [3]. TAK-242 selectively inhibits the interaction between TLR4 and its adaptor molecules, TIRAP and TRAM, via the TLR4 intracellular Cys747 residue, thereby inhibiting TLR4 downstream signaling events [18]. Here we tested the roles of TGFβ2 and TLR4 inhibition on A20 expression. Treatment with TAK-242 alone increases A20 mRNA expression when compared against control, TGFΒ2, and combination treatments (Figure 2). There was no significant difference in A20 mRNA expression after TGFβ2 treatment compared to control.

### 2.3. TGFβ2 Treatment Decreases the Expression of A20 in Primary TM Cells

**Figure 3:**
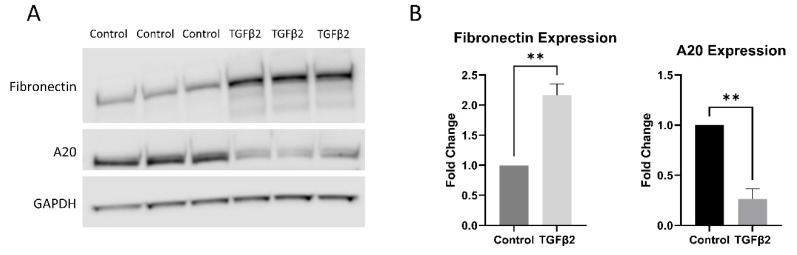
TGFβ2 increases fibronectin expression, while decreasing A20 expression, in primary human TM cells. (**A**) Primary human TM cells were treated with TGFβ2 for 48 h, after which total fibronectin and A20 expression levels were measured by Western blot, representative blot shown. (**B**) Both fibronectin and A20 expression were quantified by densitometry relative to GAPDH. Treatment with TGFβ2 increases fibronectin expression in primary human TM cells, while at the same time decreasing the expression of A20. Statistical significance was determined by Student’s *t*-test, with *p* < 0.05 considered statistically significant. Error bars show SEM. ** = *p* ≤ 0.01.

Having shown that TGFβ2 did not alter the amount of A20 message present in TM cells, we next tested the effect of TGFβ2 on A20 at the protein level in TM cells. It is known that TGFβ2 lowers A20 expression in other cell types [13]. After 48 h of TGFβ2 treatment, TM cells exhibit a fibrotic response by increasing expression of fibronectin, while at the same time decreasing expression of A20 (Figure 3).

### 2.4. A20 Overexpression Rescues the Fibrotic Response in Primary TM Cells

**Figure 4:**
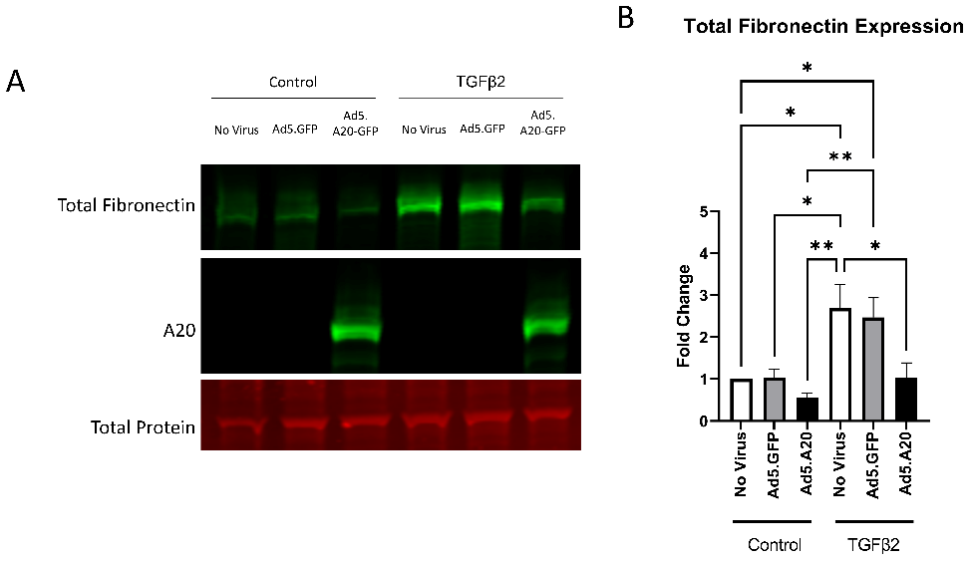
TGFβ2-induced fibronectin expression is attenuated with A20 overexpression. Primary TM cells were treated with the indicated Ad.5 virus and then with TGFβ2 for 48 h. (**A**) Representative Western blot showing fibronectin and A20 expression, with the total protein stain used for normalization. (**B**) Fibronectin expression was greatly induced by the addition of TGFβ2, and A20 overexpression blocked TGFβ2-induced fibronectin expression. Statistical significance was calculated by one-way ANOVA with Tukey post-hoc analysis, with *p* < 0.05 considered statistically significant. Error bars show SEM. * = *p* ≤ 0.05, ** = *p* ≤ 0.01.

**Figure 5:**
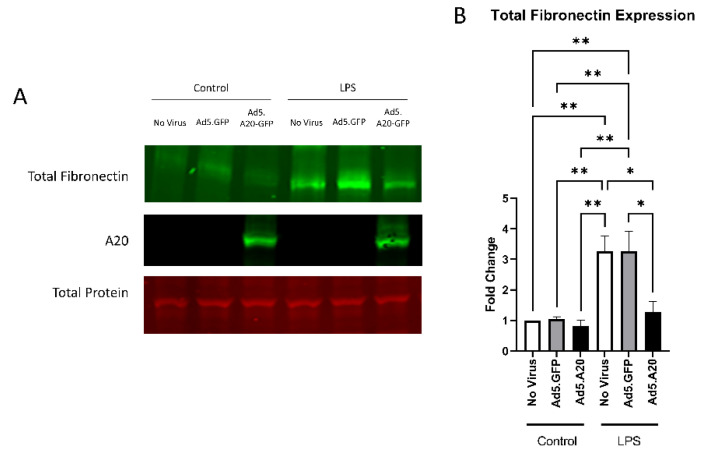
LPS-induced fibronectin expression is attenuated with A20 overexpression. Primary TM cells were treated with the indicated Ad.5 virus and then with LPS for 48 h. (**A**) Representative Western blot showing fibronectin and A20 expression, with the total protein stain used for normalization. (**B**) Fibronectin expression was greatly induced by the addition of LPS, and A20 overexpression blocked LPS-induced fibronectin expression. Statistical significance was calculated by one-way ANOVA with Tukey post-hoc analysis, with *p* < 0.05 considered statistically significant. Error bars show SEM. * = *p* ≤ 0.05, ** = *p* ≤ 0.01.

**Figure 6:**
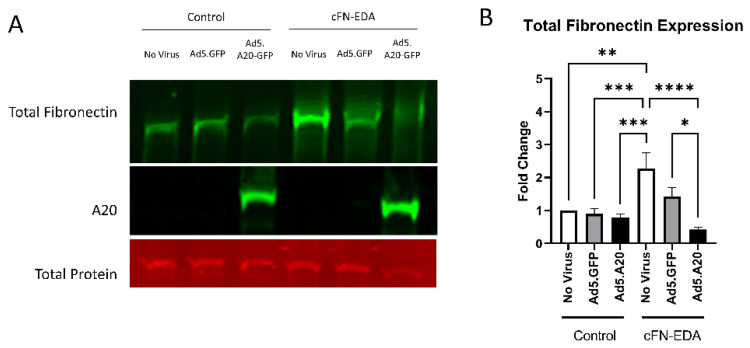
cFN-EDA-induced fibronectin expression is attenuated with A20 overexpression. Primary TM cells were treated with the indicated Ad.5 virus and then with cFN-EDA for 48 h. (**A**) Representative Western blot showing fibronectin and A20 expression, with the total protein stain used for normalization. (**B**) Fibronectin expression was greatly induced by the addition of cFN-EDA, and A20 overexpression blocked cFN-EDA-induced fibronectin expression. Statistical significance was calculated by one-way ANOVA with Tukey post-hoc analysis, with *p* < 0.05 considered statistically significant. Error bars show SEM. * = *p* ≤ 0.05, ** = *p* ≤ 0.01, *** = *p* ≤ 0.001, **** = *p* ≤ 0.0001.

Knowing that TGFβ2 decreased the expression of A20, we next tested if overexpressing A20 would rescue the fibrotic response in TM cells. For these studies, we utilized an Ad5 viral vector containing A20 transgene with a GFP tag to transduce primary TM cells. Ad5.GFP vector and untreated TM cells without a viral vector were used as controls. After transduction overnight, cells were treated with either TGFβ2, LPS (a known classical activator of TLR4), or cFN-EDA (a known DAMP and activator of TLR4), and lysates were collected and immunoblotted for either fibronectin to assess the fibrotic response or for A20 to confirm overexpression. In all conditions, A20 overexpression rescued the fibrotic response induced by TGFβ2 (Figure 4), LPS (Figure 5), and cFN-EDA (Figure 6) when compared to control treated cells. 

### 2.5. B6.EDA^+/+^ Mice Cyclically Express Higher Levels of A20 RNA in the TM Region

**Figure 7:**
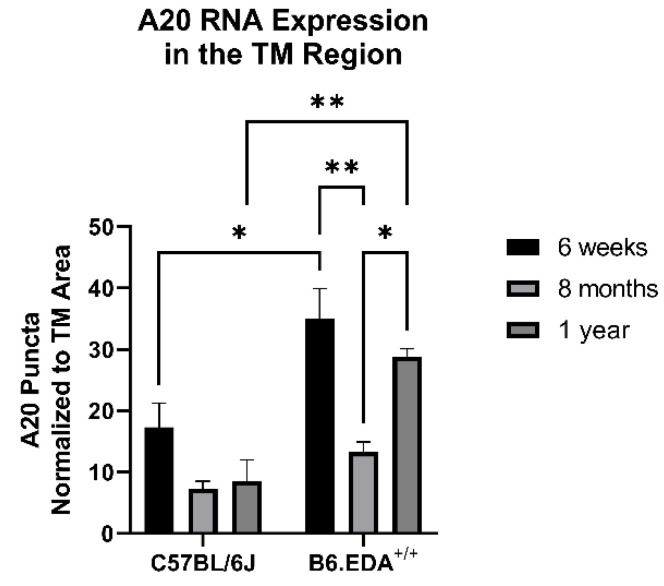
A20 RNA expression has a cyclic relationship with age in B6.EDA^+/+^ mice versus control C57BL/6J mice. Mouse eyes (*n* = 3 for each timepoint and mouse strain) were harvested from age-matched control C57BL/6J mice and B6.EDA^+/+^ mice. In situ hybridization for A20 was performed, and RNA puncta were counted in the TM outflow region and normalized to the TM area. A20 expression was higher in B6.EDA^+/+^ mice at 6 weeks and 1 year of age, but not at 8 months, suggesting a cyclic relationship between A20 and age in B6.EDA^+/+^ mice. Importantly, no change was seen in A20 RNA expression in the control C57BL/6J mice across any timepoints. Statistical significance was calculated by two-way ANOVA with Tukey post-hoc analysis, with *p* < 0.05 considered statistically significant. Error bars show SEM. * = *p* ≤ 0.05, ** = *p* ≤ 0.01.

Since A20 overexpression rescued the fibrotic response in primary TM cells, we next tested the role of A20 in our in vivo mouse model of glaucoma: B6.EDA^+/+^ mice, which constitutively express the known DAMP FN-EDA and develop ocular hypertension and TM damage [5,19]. We utilized in situ hybridization to probe for A20 RNA expression in age-matched control C57BL/6J mice and B6.EDA^+/+^ mice. At 6 weeks and 1 year of age, A20 RNA expression was increased in the TM region of B6.EDA^+/+^ mice compared to control mice (Figure 7). However, at 8 months of age, we did not observe a difference in A20 RNA expression. This implies that there is a cyclic relationship between A20 levels in regard to age. Importantly, there was no change in A20 RNA levels in any of the control mice across any of the timepoints.

## 3. Discussion

Even though glaucoma is the second leading cause of blindness worldwide, the molecular mechanisms for its progression remain to be fully elucidated [20]. It is understood that changes in the ECM in the TM can alter the ability of aqueous humor to properly drain from the anterior chamber [2]. Specifically, profibrotic TGFβ2 signaling has been suggested to be involved in glaucomatous damage to the TM [3]. We have built upon this work by exploring the mechanism by which TGFβ2–TLR4 signaling crosstalk is responsible for the regulation of ECM and fibrogenesis in the TM as well as in the development of ocular hypertension [3,21]. We have shown that endogenous ligands of TLR4, such as the fibronectin isoform FN-EDA, activate TLR4 and augment TGFβ signaling and downstream fibrotic responses [3]. This activation of the fibrotic response leads to an accumulation of more FN-EDA, triggering a feedforward loop and thus furthering the progression of fibrosis within the TM. We have shown that crosstalk between TGFβ2 and the transcription factor nuclear factor kappa beta (NF-κB) signaling pathways in the TM contributes to glaucomatous ocular hypertension, as NF-κB is necessary for TGFβ2-induced ECM production [6]. Since NF-κB is necessary for the fibrotic response, we investigated how a suppressor of NF-κB, A20, may be able to rescue the fibrotic response within the TM. Importantly, the A20 gene has recently been shown to be downregulated in human TM cells that were made to have constitutively active αvβ3 integrin [22]. The activation of this integrin is suggested to contribute to the fibrotic-like changes observed in primary open-angle glaucoma, therefore A20’s diminished presence may be contributing to the increased fibrotic response seen in glaucoma. Additionally, changes in the expression of A20 have been identified within the retina of human donor eyes confirmed to have a diagnosis of glaucoma [12]. These data support the role of A20 in attenuating a fibrotic response within the TM.

Since A20 expression is known to be suppressed by TGFβ2 signaling, we examined how A20 was being affected by the addition of TGFβ2 both at the message and protein level [13]. We found that TGFβ2 alone did not affect the message of A20, yet inhibition of the crosstalk pathway on TLR4 with the selective inhibitor TAK-242 did increase A20 message. This may be a mechanism whereby cells are able to “store” A20 in reserve when it is not directly being accessed, and then have it more readily available when insults are presented to the cells. This is supported by the fact that A20 showcases a rapid resurgence in cells upon NF-κB activation [8]. At the protein level, similar to other cell types, TGFβ2 did decrease the amount of A20 expressed within TM cells, alongside a higher level of fibronectin expression.

After determining that TGFβ2 and the TLR4 pathways both altered A20 levels in TM cells, we investigated how overexpression of A20 affected the TGFβ2 pathway by direct addition of TGFβ2, as well as the TLR4 pathway by classical stimulation with LPS and with the DAMP FN-EDA. We demonstrated that A20 is able to rescue the fibrotic response in primary TM cells, when they are challenged by both the classical activator TGFβ2 and by TLR4 specific activators LPS and FN-EDA. These data illustrate that TGFβ2–TLR4 crosstalk is affected by A20. This insight that A20 can recue the fibrotic response is an encouraging finding as a new target to combat the buildup in fibrosis seen within glaucoma. A previous study has also shown that when A20 is overexpressed in cardiac fibroblasts and then treated with TGFβ for 48 h, the expression of the ECM components collagen I and collagen II are inhibited [23]. Similarly, downregulation of A20 expression allowed for an enhancement of collagen I and collagen III expression in cardiac fibroblasts after TGFβ treatment [23]. In relation to our work, the overexpression of A20 may be attenuating the fibrotic response by being able to bind more readily to NF-κB due to having a higher abundance of A20 that can utilize its two binding sites in the A20 promoter [8]. This idea is further supported by data showing that overexpression of A20 in pancreatic islets of Langerhans confers resistance to cytokine-mediated activation of NF-κB, and further data that revealed that airway inflammation in lungs was attenuated by A20 overexpression [10,11].

We next showed that mice that constitutively express only FN containing EDA, a known DAMP and activator of TLR4, have higher levels of A20 RNA expression compared to control mice at 6 weeks and 1 year of age. These data suggest that the constant activation of TLR4 by the DAMP FN-EDA is leading to increased A20 expression to combat the high levels of DAMP activation. However, at 8 months of age we observed a decrease in A20 expression in B6.EDA^+/+^ mice compared to 6-week- and 1-year-old mice, suggesting there is a cyclic relationship of A20 expression and age in B6.EDA^+/+^ mice. A similar phenomenon was observed when an in vitro kinase activity assay was used to measure the activity of TAK1, an upstream regulator of Smad signaling which eventually leads to the activation of fibrosis, in mice that overexpressed A20 [23]. In that study, control mice and transgenic mice that overexpressed A20 underwent aortic banding surgery to expose cardiac cells to biomechanical stress. Then TAK1 activity was measured at 1 day, 7 days, and then at 2, 4, and 8 weeks. In all cases, TAK1 activity in the mice overexpressing A20 was decreased [23]. At the same time, as both sets of mice aged, TAK1 activity peeked at 4 weeks and then decreased in activity. Interestingly, while the activity of TAK1 was lowered by A20 overexpression, the total amount of TAK1 protein was unchanged in both sets of mice. This implies that A20 may be able to stop the activation of TAK1 but not alter the overall amount of the protein present. Future work will be needed to determine if A20 is altering the amount of NF-κB activation or its total levels in the TM. However, it is known that A20 gene expression can be downregulated in TM cells, and changes in the expression of A20 have been associated with glaucoma [12,22]. Specifically, immortalized trabecular meshwork cell lines with constitutively active αvβ3 integrin, activation of which could contribute to fibrosis in POAG, had downregulated expression of A20 compared to wild-type control cells [22]. In glaucomatous human retinas, A20 expression has also been found to be downregulated [12]. Overall, these studies show that A20 is a promising candidate for reducing the fibrotic response brought about by glaucomatous TM damage.

## 4. Materials and Methods

### 4.1. Experimental Animals

All experiments were conducted in compliance with the ARVO Statement for the Use of Animals in Ophthalmic and Vision Research and approved by the University of Wisconsin Madison (UW-Madison; Madison, WI, USA) Institutional Animal Care and Use Committee (IACUC) guidelines and regulations. The generation of B6.EDA^+/+^ mice has previously been described [24]. Briefly, B6.EDA^+/+^ mice were generated to contain spliced sites at both splicing junctions of the EDA exon and therefore constitutively express only FN containing EDA. Age-matched C57BL/6J mice were used as controls and obtained from the Jackson Laboratory (Bar Harbor, ME, USA). All animals were housed in the UW-Madison vivarium. At each timepoint (6 weeks, 8 months, and 1 year) *n* = 3 mice per experimental group were utilized.

### 4.2. Primary Human TM Cell Culture and Characterization

TM tissue was excised from human donor eyes obtained from the Lions Eye Bank of Wisconsin within 24 h post-mortem and grown in DMEM + 20% FBS supplemented with 1% Penn/Strep and 1% L-glutamine. Once TM cells had grown off the trabecular tissue and reached confluence in the culture dish (~3 months), cells were expanded into culture flasks in DMEM + 10% FBS supplemented with 1% Penn/Strep and 1% L-glutamine and subsequently plated for characterization by Western blotting or immunocyto chemistry (ICC). After reaching confluence, cells were treated with 100 nM dexamethasone (D4902, Sigma, St. Louis, MO, USA) for either 1 week (ICC analysis) or 96 h (Western blot analysis). Coverslips were probed for myocilin expression (1:100, MABN866, Millipore, Burlington, MA, USA) and phalloidin (1:400, A34055, Invitrogen, Waltham, MA, USA), using standard ICC techniques, to evaluate CLAN formation with F-actin labeling. Western blots were probed for myocilin expression (1:2000, MABN866, Millipore, Burlington, MA, USA) using standard techniques and normalized against GAPDH (1:5000, 3683S, Cell Signaling, Danvers, MA, USA) as a loading control. Statistical significance was determined by Student’s *t*-test, with *p* < 0.05 considered statistically significant.

### 4.3. Ad.5 Overexpression and TGFβ2, LPS, and cFN-EDA Treatment

Adenovirus 5 (Ad5) viral vector expressing human A20 with a green fluorescent protein (GFP) reporter (referred to throughout as Ad5.A20) (Vector Biolabs, Malvern, PA, USA) was used to overexpress A20. Ad5.GFP virus (Vector Biolabs, Malvern, PA, USA) was used as a negative control. Briefly, primary NTM cells were grown to 90% confluency, after which they were rinsed twice with serum-free media and either treated with Ad5.GFP or Ad5.A20 overnight, each at a multiplicity of infection (MOI) of 50. Supplementary Figure S1 shows that an MOI of 50 provided a high transduction rate in primary NTM cells. Cells were also maintained in serum-free media with no virus added as an additional control. The next morning, the viral media was removed and replaced with fresh serum-free media, and cells were then treated with either TGFβ2 (5 ng/mL, 302-B2-002, Novus, Centennial, CO, USA), LPS (100 ng/mL, L3024-5MG, Sigma, St. Louis, MO, USA), or cFN-EDA (10 µg/mL, F2518, Sigma, St. Louis, MO, USA) for 48 h. After 48 h, lysates were harvested, and Western blot experiments were performed as described below.

Importantly, cellular fibronectin containing FN-EDA was isolated from human foreskin fibroblast (F2518; Sigma, St. Louis, MO, USA) and reconstituted with sterile phosphate-buffered saline solution (PBS) to a stock concentration of 1 mg/mL. Precautions were taken to avoid repeated thawing/freezing steps.

### 4.4. Western Blotting

All Western blot studies were performed as stated unless otherwise noted. Briefly, NTM cell strains were treated as stated above for 48 h, after which whole cell lysate was collected from each condition. Cells were rinsed twice with ice-cold PBS, and cell lysates were extracted using lysis buffer (RIPA buffer, Thermo Fisher, Waltham, MA, USA) with protease and phosphatase inhibitors added before use (11,836,170,001 and 4,906,837,001, Sigma, St. Louis, MO, USA). Lysates were then centrifuged at 14,000× *g* for 10 min at 4 degrees Celsius, after which the supernatant was removed to a clean tube. Protein concentration was determined by the Pierce BCA protein assay (23225, Thermo Fisher, Waltham, MA, USA). Samples were mixed with 4X loading buffer (928-40004, Li-Cor, Lincoln, NE, USA), with each sample containing 7.5 µg of protein. Samples were heated at 70 degrees Celsius for 10 min, followed by separation using a 4–12% gradient gel (NW04125BOX, Invitrogen, Waltham, MA, USA). Proteins from electrophoresed gels were transferred to polyvinylidene (PVDF) membranes (IB24002, Thermo Fisher, Waltham, MA, USA) using the iBlot2 system (IB21001, Thermo Fisher, Waltham, MA, USA). After transfer, membranes were dried and then stained for total protein (926-11010, LiCor, Lincoln, NE, USA) expression as a loading control according to the manufacturer’s directions on the Licor OdysseyCLx system (LiCor, Lincoln, NE, USA). Total protein stain was then removed, and membranes were blocked using Intercept TBS blocking buffer (927-66003, LiCor, Lincoln, NE, USA) for 1 h at room temperature. Membranes were immunolabeled overnight at 4 degrees Celsius with primary antibodies diluted in Intercept TBS antibody diluent (927-66003, LiCor, Lincoln, NE, USA): rabbit anti-fibronectin (AB1945, Millipore, Burlington, MA, USA) dilution 1:5000 and rabbit anti-A20 (ab92324, Abcam, Waltham, MA, USA) dilution 1:1000. The next day, membranes were incubated for 1 h at room temperature with IRDye^®^ 800CW goat anti-rabbit IgG secondary antibody (926-32211, LiCor, Lincoln, NE, USA) at 1:20,000 diluted in Intercept TBS antibody diluent. Membranes were imaged on a Licor OdysseyCLx system.

Each experiment was repeated a minimum of three times for each individual primary NTM cell strain. Densitometry analysis of Western immunoblot images was used to determine changes in protein content after treatment. Band intensity for FN and A20 was measured using Image Studio Lite (version 5.2.5, LiCor, Lincoln, NE, USA). Each target protein densitometry value was normalized against its corresponding total protein value, and fold change was compared to control and represented as the mean ± SEM.

For groups of 2, statistical significance was determined by Student’s *t*-test, with *p* < 0.05 considered statistically significant. For groups of 3 or more, statistical significance was calculated by one-way ANOVA with Tukey post-hoc analysis, with *p* < 0.05 considered statistically significant.

### 4.5. RT-PCR

After 48 h of treatment as described above with TGFβ2 and/or TAK-242 (15 µM, also known as CLI-095; InvivoGen, San Diego, CA, USA) or none as a control, primary human TM cells had RNA extracted using the RNeasy Plus Mini Kit (Qiagen, Valencia, CA, USA) according to the manufacturer’s directions. Samples were then reverse-transcribed to cDNA (Bio-Rad iScript cDNA synthesis kit; Bio-Rad Laboratories, Hercules, CA, USA). Each PCR reaction was run with the Advantage 2 RT-PCR kit (Takara-Bio, Mountain View, CA, USA) and contained: 20 µL PCR-grade H_2_O, 2.5 µL 10X Advantage 2 PCR buffer, 0.5 µL 50X dNTP mix, 0.5 µL Advantage 2 polymerase mix, 0.2 µL forward primer (100 µM), 0.2 µL reverse primer (100 µM), and 1 µL cDNA template (150 ng/µL). Primers used in the PCR reactions were human A20 (accession number NM_001270508.2 FWD 5′-CCCTGGAAAGCCAGAAGAAA-3′, REV 5′-GCTGCTATAGCCGAGAACAA-3′) and human glyceraldehyde-3-phosphate dehydrogenase (GAPDH) (accession number NM_002046.7 FWD 5′-CTTTGGTATCGTGGAAGGACTC-3′, REV 5′-AGTAGAGGCAGGGATGATGT-3′). Samples were run on a 1.5% agarose gel and visualized with GelRed nucleic acid stain (Biotium, Hayward, CA, USA) using an Azure c300 imaging system (Azure Biosystems, Dublin, CA, USA). Statistical significance was calculated by 1-way ANOVA with Tukey post-hoc analysis, with *p* < 0.05 considered statistically significant.

### 4.6. In Situ Hybridization

To detect A20 mRNA, mouse mRNA probes (#426481) were purchased from Advanced Cell Diagnostics (Newark, CA, USA). Incubation and detection of probes was performed according to the manufacturer’s instructions. Mouse eyes were harvested from age-matched control C57BL/6J mice and B6.EDA^+/+^ mice, after which they were fixed in 4% PFA and cryosectioned. Slides were baked at 60 degrees Celsius for 30 min, washed with ddH_2_O, baked at 60 degrees Celsius for 60 min, and pretreated with hydrogen peroxide. Antigen retrieval was performed in antigen retrieval buffer for 5 min at 85 degrees Celsius. Slides were laid flat during the antigen retrieval step to diminish the chance of the section detaching. Hydrophobic borders were created around sections with an ImmEdge pen (H-4000, Vector Laboratories, Burlingame, CA, USA), after which proteinase K was applied for 30 min at 40 degrees Celsius in a humidified chamber. Sections were rinsed, and the A20 mRNA probe was added for 2 h, followed by a series of rinses and addition of amplification reagents, each for 30 min. Signal was amplified with the TSA Cy3 reagent (#SAT704A001EA, Akoya, Menlo Park, CA, USA). Sections were imaged on a Nikon A1RS confocal microscope (Nikon, Melville, NY, USA), with the resulting Z-stacks of the outflow region merged into a maximum intensity projection within the NIS-Elements software (version 5.20.01, Nikon, Melville, NY, USA).

For quantification of mRNA puncta in the TM, counting was performed using ImageJ (version 1.53c, National Institutes of Health, USA) on the maximum intensity projections. The phase contrast images were used to define the entire TM outflow region with the polygon selection tool. The RNA puncta in this region were then inverted in color and counted in ImageJ within the defined TM region. This puncta count was then normalized to the total area of that eye’s TM outflow region. Supplementary Figure S2 shows an example of how this quantification was performed. Statistical significance was calculated by 2-way ANOVA with Tukey post-hoc analysis, with *p* < 0.05 considered statistically significant.

### 4.7. Statistical Analysis

All statistical analyses were performed as described in each method above utilizing GraphPad Prism 9 (version 9.3.1, San Diego, CA, USA). The *p*-values represented by asterisks are as follows: * = *p* ≤ 0.05; ** = *p* ≤ 0.01; *** = *p* ≤ 0.001; **** = *p* ≤ 0.0001.

## Data Availability

The data that support the findings of this study are available from the corresponding author upon reasonable request.

## Supplementary Materials

The following supporting information can be downloaded at: https://www.mdpi.com/article/10.3390/ijms23041928/s1.
