# Supplementary figures and images for: A20 Attenuates the Fibrotic Response in the Trabecular Meshwork
Source: Int J Mol Sci. 2022 Feb 9;23(4):1928. doi: 10.3390/ijms23041928 (PMC8875798; doi:10.3390/ijms23041928)

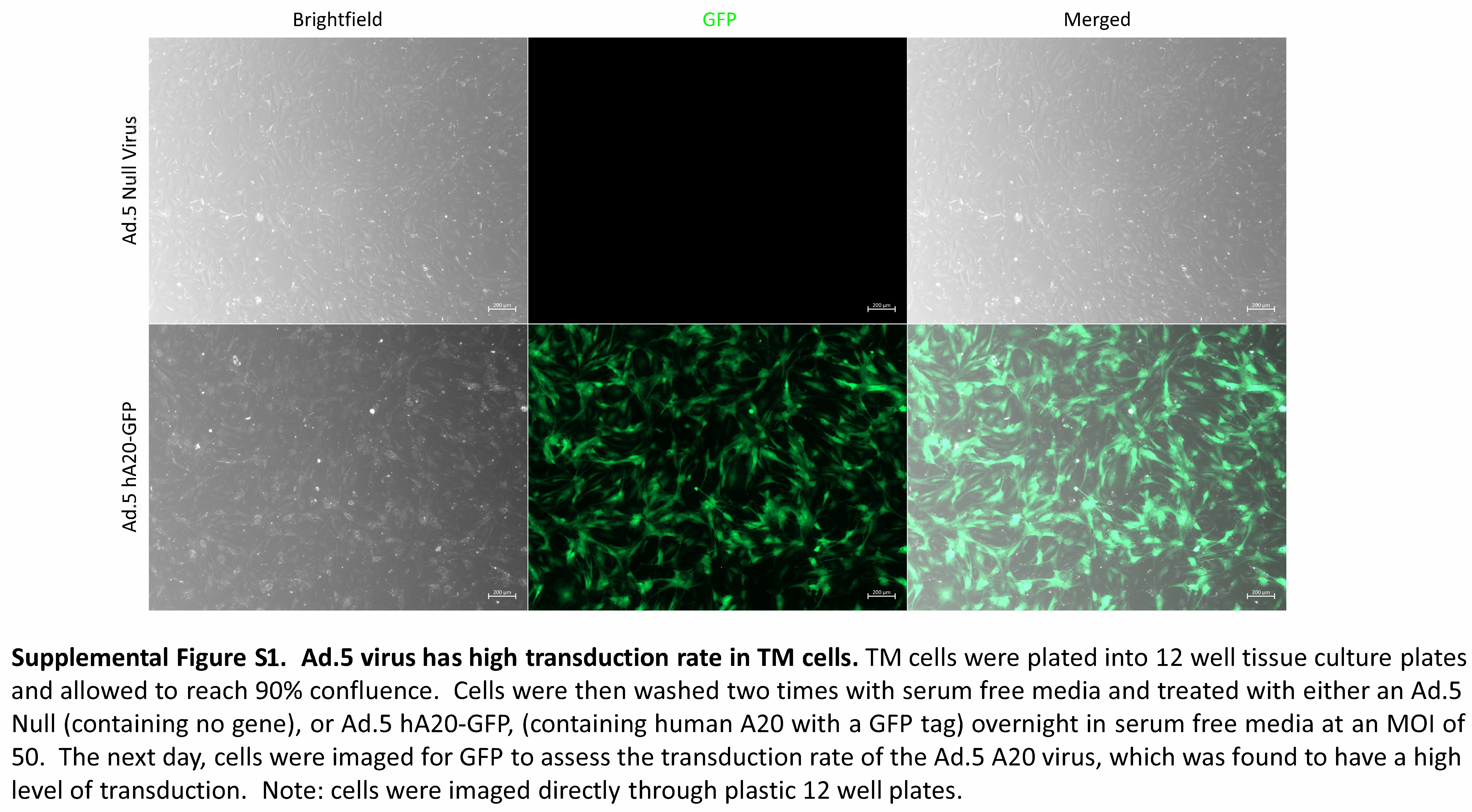

Supplement: Supplementary file 1 [file ijms-23-01928-s001.zip › Supplemental Figure S1.tif]

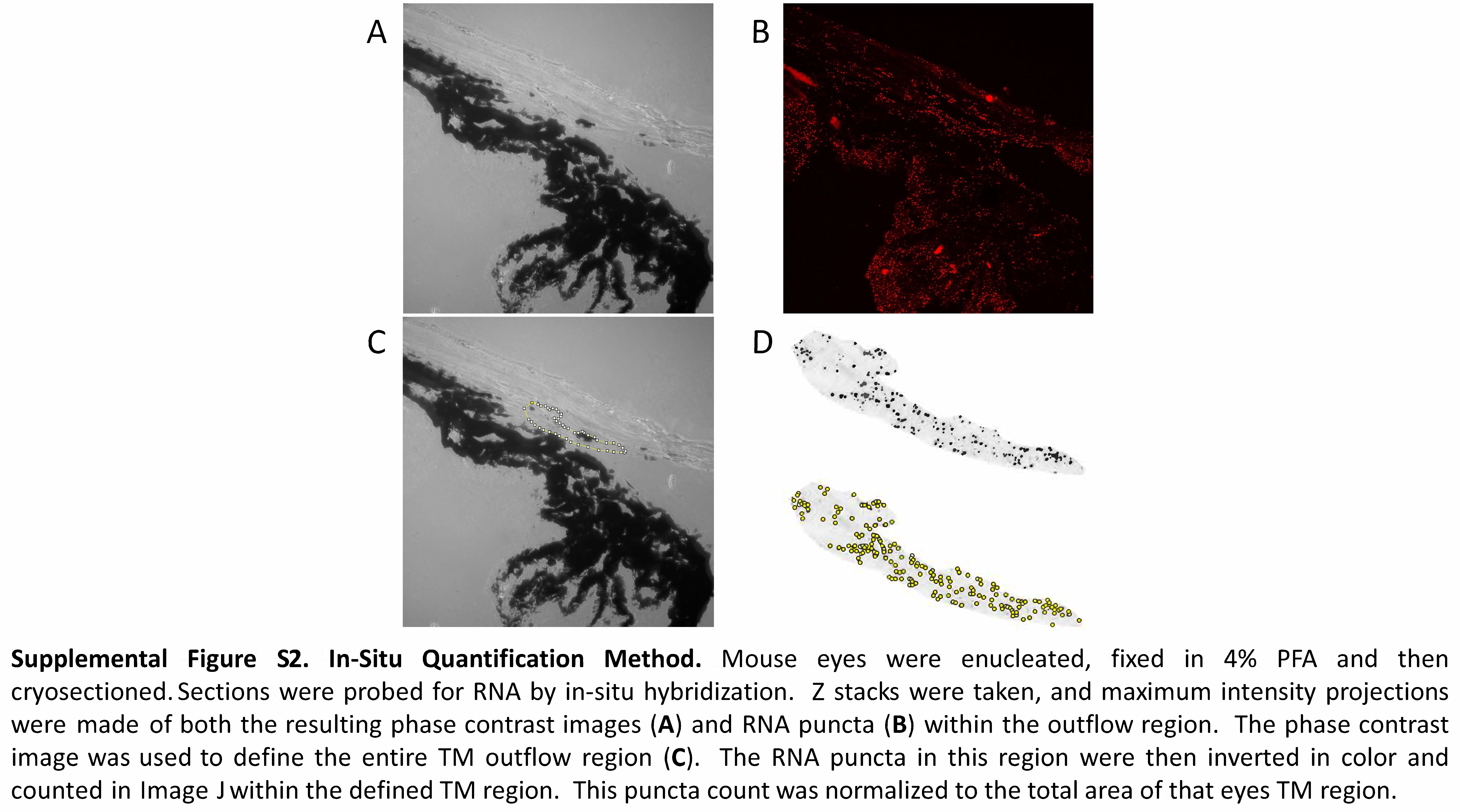

Supplement: Supplementary file 1 [file ijms-23-01928-s001.zip › Supplemental Figure S2.tif]
